# Optimising Deep Learning at the Edge for Accurate Hourly Air Quality Prediction

**DOI:** 10.3390/s21041064

**Published:** 2021-02-04

**Authors:** I Nyoman Kusuma Wardana, Julian W. Gardner, Suhaib A. Fahmy

**Affiliations:** 1 School of Engineering, University of Warwick, Coventry CV4 7AL, UK; J.W.Gardner@warwick.ac.uk (J.W.G.); suhaib.fahmy@kaust.edu.sa (S.A.F.); 2 Computer, Electrical and Mathematical Sciences and Engineering, King Abdullah University of Science and Technology (KAUST), Thuwal 23955, Saudi Arabia; 3 Department of Electrical Engineering, Politeknik Negeri Bali, Badung, Bali 80364, Indonesia

**Keywords:** air quality prediction, PM_2.5_ prediction, deep learning, post-training quantisation, edge computing

## Abstract

Accurate air quality monitoring requires processing of multi-dimensional, multi-location sensor data, which has previously been considered in centralised machine learning models. These are often unsuitable for resource-constrained edge devices. In this article, we address this challenge by: (1) designing a novel hybrid deep learning model for hourly PM_2.5_ pollutant prediction; (2) optimising the obtained model for edge devices; and (3) examining model performance running on the edge devices in terms of both accuracy and latency. The hybrid deep learning model in this work comprises a 1D Convolutional Neural Network (CNN) and a Long Short-Term Memory (LSTM) to predict hourly PM_2.5_ concentration. The results show that our proposed model outperforms other deep learning models, evaluated by calculating RMSE and MAE errors. The proposed model was optimised for edge devices, the Raspberry Pi 3 Model B+ (RPi3B+) and Raspberry Pi 4 Model B (RPi4B). This optimised model reduced file size to a quarter of the original, with further size reduction achieved by implementing different post-training quantisation. In total, 8272 hourly samples were continuously fed to the edge device, with the RPi4B executing the model twice as fast as the RPi3B+ in all quantisation modes. Full-integer quantisation produced the lowest execution time, with latencies of 2.19 s and 4.73 s for RPi4B and RPi3B+, respectively.

## 1. Introduction

Edge computing refers to the deployment of computation closer to data sources (edge) [1], rather than more centrally as is the case with cloud computing. It can address latency, privacy and scalability issues faced by cloud-based systems [2,3]. In terms of latency, moving computation closer to the data sources decreases end-to-end network latency. In terms of privacy, computation performed at the edge or at a local trusted edge server prevents data from leaving the device, potentially reducing the chance for cyber-attacks. In terms of scalability, edge computing can avoid network bottlenecks at central servers by enabling a hierarchical architecture of edge nodes [4]. Moreover, edge computing can address energy-aware and bandwidth saving applications [5].

For data processing and information inference, it is also possible to embed intelligence at edge devices, which can be enabled by machine learning (ML) algorithms [6,7]. Deep learning [8], a subset of Machine Learning, can be implemented on edge devices, such as mobile phones, wearables and the Internet of Things (IoT) nodes [9,10]. Deep learning is more resilient to noise and able to deal with non-linearity. Instead of relying on hand-crafted features, deep learning automatically extracts the best possible features during its training phase. During training, the deep neural network architecture can extract very coarse low-level features in its first layer, recognise finer and higher-level features in its intermediate layers and achieve the targeted values in the final layer [11].

Efficient deep learning design (e.g., deep neural networks) for embedded devices can be achieved by optimising both algorithmic (software) and hardware aspects [11]. At the algorithmic level, two methods can be implemented, namely model design and model compression [4]. In model design, researchers focus on designing deep learning models with a reduced number of parameters. This results in reduced memory size and latency, while trying to maintain high accuracy. In model compression, models are adapted for edge deployment by applying a number of different techniques on a trained model, such as parameter quantisation, parameter pruning and knowledge distillation. Parameter quantisation is a conversion technique to reduce model size with minimal degradation in model accuracy. Parameter pruning eliminates the least essential values in weight tensors. This method is related to the dropout technique [12]. Knowledge distillation [13] creates a smaller deep learning model by mimicking the behaviour of a larger model. It can be realised by training the smaller model using the outputs obtained from the larger model. At the hardware level, the training and inferencing processes of deep learning models can be accelerated by the computation power of server-class central processing units (CPUs), graphics processing unit (GPUs), tensor processing units (TPUs), neural processing units (NPUs), application-specific circuits (ASICs) and field-programmable gate arrays (FPGAs). Deep learning accelerators with diversity of layers and kernels built from custom low density FPGAs can provide high-speed computation while maintaining reconfigurability [14]. Both ASICs and FPGAs are generally more energy-efficient than conventional CPUs and GPUs [4].

Deep learning at the edge can be applied for air pollution prediction. Air pollution exposure causes negative impacts on human health [15,16] and economic activities [17]. Among many air pollutants, particulate matter (PM) harms the human respiratory system, as it may enter into the human respiratory tract or even the lungs through inhalation [18,19]. Particulate matter can be in the form of PM_2.5_ (particulate matter with diameter less than 2.5 μm, or fine particles) and PM_10_ (diameter less than 10 μm, or inhalable particles) [20]. It may lead to lung cancer [20], affect cardiovascular diseases [21] and even result in death [22]. Particulate matter causes premature death, and it is considered as responsible for 16% of global deaths [23]. The complex mixture of particulate matter and other gases like ozone was recorded to be associated with an all-cause death rate of up to 9 million in 2015 [24]. In this connection, building a forecasting system based on hourly air quality prediction plays an important role in health alerts [25].

Many works on PM_2.5_ prediction considered only the performance evaluation by comparing predicted values to the dataset for accuracy. Our work aims to extend this body of work around deep learning models for air quality monitoring by analysing the deployment of these models to edge devices. In this work, our main contributions are:

(1) designing a novel hybrid deep learning model for PM_2.5_ pollutant level prediction based on an available dataset;
(2) optimising the obtained model to a lightweight version suitable for edge devices; and
(3) examining model performance when running on the edge devices.

We implement post-training quantisation as a part of the algorithmic-level optimisation. This technique compresses model parameters by converting floating-point numbers to reduced precision numbers. Quantisation can improve CPU and hardware accelerator latencies and potentially reduce the original deep learning model size.

The remainder of this paper is structured as follows. Section 2 summarises the related work and clarifies our originality. Section 3 explains some of the basic theories related to this research. Section 4 describes the dataset and the required preprocessing, as well as defining our proposed deep learning model and gives a brief overview of the edge devices used in this work. Section 5 presents the results of our proposed model in terms of prediction accuracy and explains the model optimisation results for the selected edge devices. Section 6 offers conclusions and discusses future work.

## 2. Related Work

Various work been published in the last few years around the use of deep learning for air quality prediction. Navares and Aznarte [26] implemented Long Short-Term Memory (LSTM) to predict PM_10_ and other air pollutants. They demonstrated a Recurrent Neural Network (RNN) that can map input sequences to output sequences by including the past context into its internal state, making it suitable for time-series problems. However, as the time series grows, relevant information occurs further in the past making RNNs unable to connect suitable information. Moreover, RNNs suffer from the vanishing gradient problem due to cyclic loops.

LSTMs, a variation of RNNs, are capable of learning long-term dependencies and are able to deal with vanishing gradients. Li et al. [27] predicted hourly PM_2.5_ concentration by using an LSTM model. The authors combined historical air pollutant data, meteorological data, and time stamp data. For one-hour predictions, the proposed LSTM model outperformed other models such as the spatiotemporal deep learning (STDL), time-delay neural network (TDNN), autoregressive moving average (ARMA) and support vector regression (SVR) models. Xayasouk et al. [28] implemented LSTM and Deep Autoencoder to predict 10-day of PM_2.5_ and PM_10_ concentrations. By varying the input batch size and recording the total average of the model performances, the proposed LSTM model was more accurate than the DAE model. Seng et al. [29] used LSTM model to predict air pollutant data (PM_2.5_, CO, NO_2_, O_3_, SO_2_) at 35 monitoring stations in Beijing. They proposed a comprehensive model called multi-output and multi-index of supervised learning (MMSL) based on spatiotemporal data of present and surrounding stations. The effectiveness of the proposed model was compared to the existing time series model (Linear Regression, SVR, Random Forest and ARMA) and baseline models (CNN-LSTM and CNN-Bidirectional RNN). Xu et al. [30] proposed a framework called HighAir. This framework used hierarchical graph neural network based on encoder-decoder architecture. Both encoder and decoder consist of LSTM network. Other works based on LSTMs are also reported in [31,32].

Other researchers have also proposed hybrid deep learning models. Zhao et al. [33] compared ANN, LSTM and LSTM-Fully Connected (LSTM-FC) models to predict PM_2.5_ concentrations. They found that LSTM-FC produced better predictive performance. Their model consists of two parts. In the first, the LSTM was applied to model the local PM_2.5_ concentrations. In the second, the fully connected network was used to capture the spatial dependencies between the central station and neighbour stations. The combination of CNN and LSTM models have also been actively explored [18,34,35,36]. CNN-LSTM may improve the accuracy for PM_2.5_ prediction, as reported by Li et al. [37], where the authors implemented a 1D CNN to extract features from sequence data and used LSTM to predict future values. In many real problems, input data may come from many resources, constructing spatiotemporal dependencies as explained by Qi et al. [34]. Gated Recurrent Units (GRUs), another variant of RNNs, have also been applied to PM_2.5_ prediction. Tao et al. [38] combined a one-dimensional CNN with bi-directional GRU to forecast PM_2.5_ concentration. They examined attributes in the dataset to find the best input features for the proposed model and evaluated the model performance based on mean absolute error (MAE), root mean square error (RMSE) and symmetric mean absolute percentage error (SMAPE). Powered by AI cloud computing to interpret multimode data, a new framework based on CNN-RNN was proposed by Chen et al. [39] to predict PM_2.5_ values. The framework consists of input preprocessing stages, CNN encoder, RNN-based learning network and CNN Decoder. The input model considered the spatiotemporal factor in the form of 4D sequence data of heat maps.

Various deep learning optimisation techniques have been proposed recently. Even though the selected case studies in these works might not be related to air quality prediction, we review some of them as follows. Post-trained model size can be reduced by quantising weights and activation function, without retraining the model. This method is called the *post-training quantisation* [40]. Banner et al. [40] proposed 4-bit post-training quantisation for CNNs. They designed an efficient quantisation method by minimising mean-squared quantization error at the tensor level and avoiding retraining the model. Moreover, a mathematical background review for integer quantisation and its implementation on many existing pre-trained neural network models was presented by Wu et al. [41]. With 8-bit integer quantisation, the obtained accuracy either matches or is within 1% of the floating-point model. Intended for mobile edge devices, Peng et al. [42] proposed a fully-integer based quantisation method tested on an ARMv8 CPU. The proposed method achieved comparable accuracy to other state-of-the-art methods. Li and Alvarez [43] specifically proposed the integer-only quantisation method for LSTM neural network. The obtained result is accurate, efficient and fast to execute. Moreover, the proposed method has been deployed to a variety of target hardware.

To the best of our knowledge, previous work on air quality prediction has not specifically explored optimisation of models for resource-constrained edge devices. Our work aims to extend this body of work around deep learning models for air quality monitoring by analysing the deployment of these models to edge device. We implement post-training quantisation techniques to the baseline model using tools provided by TensorFlow framework [44] and evaluate the optimised model performance on Raspberry Pi boards. Table 1 summarises the aforementioned research related to air quality prediction, alongside our contribution.

## 3. Related Theory

### 3.1. One-Dimensional Convolutional Neural Network

Many articles focus on two-dimensional Convolutional Neural Network (2D CNN) models. These networks work best for image classifications problems. The same approach can be applied to one-dimensional (1D) sequences of data (time-series data). A 1D CNN model learns to extract features from time-series data and maps the internal features of the sequence. This model is very efficient to gather information from raw time-series data directly, especially from shorter (fixed-length) segments of the overall dataset.

**Figure 1:**
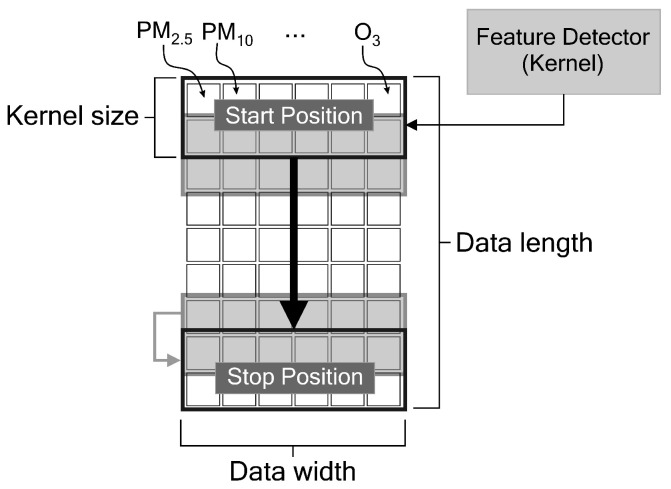
The feature detector of 1D CNN slides over time-series data.

In our case study, we extract time-series air pollutant data such as PM_2.5_, PM_10_, SO_2_, CO, NO_2_ and O_3_, and meteorological data such as temperature, air pressure, dew point, wind direction and wind speed. Figure 1 illustrates how the feature detector (or kernel) of the 1D CNN slides across the features, by assuming that our input model is only the pollutant data.

If the input data to the convolutional layer of length *n* are denoted as *x*, the kernel of size *k* as *h* and the kernel window is shifted by *s* positions, then the output *y* is defined as:

(1) $$y\left(n\right),=\left\{\begin{array}{cc} \sum_{i=0}^{k}x\left(n+i\right)h\left(i\right) & \mathrm{if}\;n=0\\ \sum_{i=0}^{k}x\left(n+i+\left(s-1\right)\right)h\left(i\right) & \mathrm{otherwise} \end{array}\right.$$

For example, if we have n=6, k=3 and s=1, then the output will be:

y(0)=x(0)h(0)+x(1)h(1)+x(2)h(2)

y(1)=x(1)h(0)+x(2)h(1)+x(3)h(2)

y(2)=x(2)h(0)+x(3)h(1)+x(4)h(2)

y(3)=x(3)h(0)+x(4)h(1)+x(5)h(2)

If it is assumed that there is no padding applied to the input data, then the length of output data *o* is given by:

(2) $$o=\lfloor \frac{n-k}{s}\rfloor +1$$

Therefore, we can find the length of *y* based on the example mentioned above, that is o=(6−3)/1+1=4.

Aside from the convolutional layer, there is a pooling layer, which downsamples the dimensions of the convolution output. There are several kinds of pooling layer, such as max pooling and average pooling. Max-pooling takes the maximum of the window, whereas average pooling takes the average value of the window. The dimensions output by the convolutional layers may be greater than one. The flattening process aims to reduce the output dimension to form a flat structure suitable for fully connected layers.

### 3.2. Long Short-Term Memory Cells

**Figure 2:**
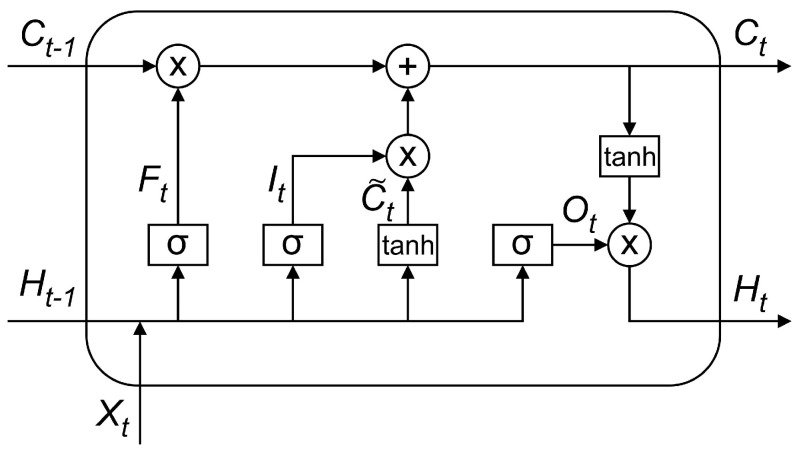
An LSTM cell structure.

Long Short-Term Memory (LSTM) [45] is a structural modification of the Recurrent Neural Network (RNN) that adds memory cells in the hidden layer so that it can be implemented to control the flow of information in time-series data. Figure 2 shows the LSTM network cell structure.

As shown in Figure 2, the network inputs and outputs on the LSTM structure are described as follows:

(3) $$F_{t}=\sigma \left(W_{f}\cdot \left[H_{t-1},X_{t}\right]+b_{f}\right)$$

(4) $$I_{t}=\sigma \left(W_{i}\cdot \left[H_{t-1},X_{t}\right]+b_{i}\right)$$

(5) $${\tilde{C}}_{t}=\tanh\left(W_{c}\cdot \left[H_{t-1},X_{t}\right]+b_{c}\right)$$

(6) $$C_{t}=F_{t}*C_{t-1}+I_{t}*{\tilde{C}}_{t}$$

(7) $$O_{t}=\sigma \left(W_{o}\cdot \left[H_{t-1},X_{t}\right]+b_{o}\right)$$

(8) $$H_{t}=O_{t}*\tanh\left(C_{t}\right)$$

(9) $$\sigma \left(x\right)=\frac{1}{1+e^{-x}}$$

(10) $$\tanh\left(x\right)=\frac{e^{x}-e^{-x}}{e^{x}+e^{-x}}$$

with $W_{f}$, $W_{i}$, $W_{c}$ and $W_{o}$ as input weights; $b_{f}$, $b_{i}$, $b_{c}$ and $b_{o}$ as biases; *t* the current time; t−1 the previous state; *X* the input; *H* the output; and *C* the status of the cell. The notation σ is a sigmoid function, which produces an input between 0 and 1. A value of 0 means not allowing any value to pass to the next stage, while a value of 1 means letting the output fully enter the next stage. The hyperbolic tangent function (tanh) is used to overcome the loss of gradients during the training process, which generally occurs in the RNN structure.

### 3.3. Error Measures

In this work, root mean square error (RMSE) and mean absolute error (MAE) are used as evaluation parameters. RMSE and MAE can be calculated using Equations (11) and (12), respectively.

(11) $$RMSE=\sqrt{\frac{\sum_{i=1}^{n}{\left(Y_{i}-{\hat{Y}}_{i}\right)}^{2}}{n}}$$

(12) $$MAE=\frac{\sum_{i=1}^{n}|Y_{i}-{\hat{Y}}_{i}|}{n}$$

where *n* is the total number of data samples, $Y_{i}$ are the measured values and ${\hat{Y}}_{i}$ are the predicted values.

### 3.4. Correlation Coefficient between Features

Correlation analysis can provide information about the correlation of two time-series features. In our work, we evaluate the time-series of air quality parameters. If time series data are vectored as $X=(x_{1},x_{2},\cdots ,x_{n})$ and there is another vector $Y=(y_{1},y_{2},\cdots ,y_{n})$, then the correlation coefficient *r* of the two vectors is calculated using the following equation:

(13) $$r=\frac{n\sum_{i=1}^{n}x_{i}y_{i}-\sum_{i=1}^{n}x_{i}\sum_{i=1}^{n}y_{i}}{\sqrt{n\sum_{i=1}^{n}{x_{i}}^{2}-{\left(\sum_{i=1}^{n}x_{i}\right)}^{2}}\sqrt{n\sum_{i=1}^{n}{y_{i}}^{2}-{\left(\sum_{i=1}^{n}y_{i}\right)}^{2}}}$$

The value of *r* in Equation (13) is the Pearson correlation coefficient. When 0<r<1, it is said that both features have positive correlations, and, when −1<r<0, they have negative correlation. A value of 0 indicates that there is no correlation between the features. When the absolute value of *r* approaches 1, then both features higher correlation. A value *r* of 1 indicates two series of data are identical.

### 3.5. TensorFlow Post-Training Quantisation

In this work, we built deep learning models using TensorFlow 2.2 framework [44]. TensorFlow provides a lightweight version called TensorFlow Lite that offers various tools to convert and run TensorFlow models on various edge devices, including mobile, embedded and IoT devices. The deep learning models were built, trained, tested and optimised on a desktop computer. From these steps, a lightweight (optimised) deep learning model was obtained. The optimised model was then deployed to the Raspberry Pi boards. To port the model, execute it and define the inputs/outputs on the Raspberry Pi boards, it is necessary to install the TensorFlow Lite Interpreter library.

**Figure 3:**
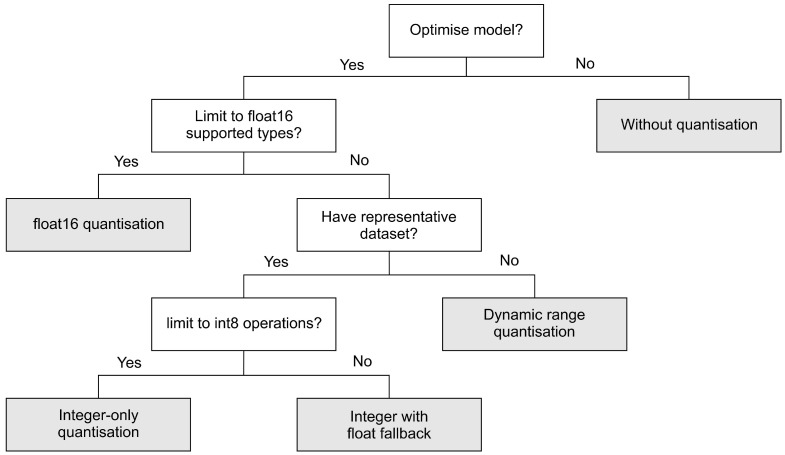
Post-training optimisation methods provided by TensorFlow [46].

TensorFlow provides tools for optimising deep learning model called the TensorFlow Model Optimisation Toolkit. Depending on the requirements of our applications, we can choose pre-optimised model, post-training or training-time optimisation tools. In this work, we focus on post-training quantisation. In post-training quantisation, the optimisation takes place after training process has been completed. There are three post-training quantisation methods provided by TensorFlow 2.2, namely *dynamic range quantisation*, *full integer quantisation* and *float16 quantisation*. Dynamic range quantisation statically quantises only the weights, from floating-point (32 bits) to integer (8 bits). During inference, weights are converted back from 8 bits to 32 bits and computed using floating-point kernels. Compared to the dynamic range quantisation, full integer quantisation offers latency improvements. Full integer quantisation supports two methods, namely *integer with float fallback* and *integer-only conversions*. The integer with float fallback means that a model can be fully integer quantised, but the execution falls back to float32 when operators do not have an integer implementation. The integer-only method is appropriate for 8-bit integer-only devices, such as microcontrollers and accelerators, e.g., EdgeTPU. In this method, the conversion fails if the model has unsupported operation. Finally, float16 quantisation converts weights to float16 (16-bit floating-point numbers). Figure 3 depicts the post-training provided by TensorFlow framework.

## 4. Materials and Methods

### 4.1. Dataset and Preprocessing

In this study, we use a dataset provided by Zhang et al. [47], which can be downloaded from the University of California, Irvine (UCI) Machine Learning Repository page. The dataset captures Beijing air quality, collected from 12 different Guokong (state controlled) monitoring sites in Beijing and its surroundings [47]. These 12 monitoring sites are Aotizhongxin, Changping, Dingling, Dongsi, Guanyuan, Gucheng, Huairou, Nongzhanguan, Shunyi, Tiantan, Wanliu and Wanshouxigong.

Regardless of the real geographical location and the ability for each monitoring site to gather both pollutant and meteorological data, we consider every monitoring site merely as a *node*. Therefore, we model a complex monitoring site as a simple node. The term node is closely associated with the end device, where the edge computing is usually executed. We are interested only in the data obtained by each node and its correlation with other nodes. We number the 12 monitoring sites as mentioned above, from Aotizhongxin as Node 1, Changping as Node 2, Dingling as Node 3, Dongsi as Node 4, etc.

**Figure 4:**
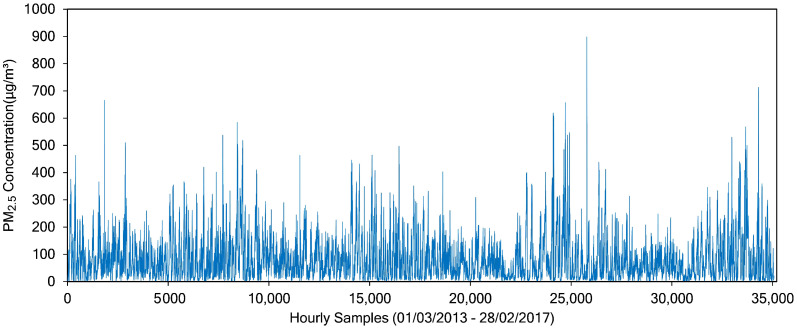
PM_2.5_ concentration gathered by Node 1 (Aotizhongxin monitoring site). This figure depicts the whole period of PM_2.5_ concentration, starting from 1 March 2013 to 28 February 2017.

There are 12 columns (features) and 36,064 rows in the dataset, collected from 1 March 2013 to 28 February 2017. Each row in the dataset is hourly data, composed of pollutant data (PM_2.5_, PM_10_, SO_2_, CO, NO_2_, and O_3_) and meteorological data (temperature, air pressure, dew point, rain, wind direction and wind speed). We split data into training data and test data. Data from 1 March 2013 to 20 March 2016 are used as training data, whereas data from 21 March 2016 to 28 February 2017 are used as test data. By using this division, there are a total of 26,784 training data and 8280 test data. In this work, we focus on predicting the PM_2.5_ concentrations. We evaluated the best model for a short-term prediction, that is 1-h particulate matter concentrations. Figure 4 shows the PM_2.5_ concentrations obtained from Node 1 (Aotizhongxin monitoring site).

Data can be numerical or categorical. The attribute of wind direction in the dataset, which is categorical data, admits 16 values: N, NNE, NE, ENE, E, ESE, SE, SSE, S, SSW, SW, WSW, W, WNW, NW and NNW. These features are label encoded. Dividing 360 degrees by 16 (number of wind directions) and applying floor rounding, we found a label for N is 360, NNE is 22, NE is 45, ENE is 67, etc. Instead of labelling N as 0, we assign it as 360. For missing values in the dataset, we filled them with the last timestamp data.

Besides labelling the categorical data and filling in missing values, we scaled the input features during the training and testing phases. Feature scaling is a method used to normalise the range of independent variables or features of data. In data processing, it is also known as data normalisation and is generally performed during the data preprocessing step. In this work, all inputs are normalised to the range of 0 and 1 (min-max scaler). The general formula for a min-max of [0, 1] is given as:

(14) $$x^{{}^{\prime }}=\frac{x-min(x)}{max(x)-min(x)}$$

### 4.2. Feature Selection

Our work aims to predict PM_2.5_. As shown in Table 2, PM_2.5_ are strongly correlated to PM_2.5_, NO_2_ and CO (with r>0.6); moderately correlated to SO_2_ (with r=0.49); and weakly correlated to O_3_ (with r=−0.15). It is also found that rain (RAIN), air pressure (PRES) and temperature (TEMP) have the weakest correlation with PM_2.5_. To obtain the optimum number of input features, only RAIN, PRES and TEMP are varied. Thus, four different combinations are obtained and the values of RMSE and MAE for each combination are recorded, as shown in Table 3. Table 3 reports the feature selection process only for Node 1. The results obtained from this step can be applied to all other nodes.

As shown in Table 3, removing rain during training (11 attributes) yielded the best performance. Thus, PM_2.5_, PM_10_, SO_2_, CO, NO_2_, O_3_, temperature, air pressure, dew point, wind direction and wind speed were selected as the input features for our model. We use the same input features for all monitoring sites.

**Figure 5:**
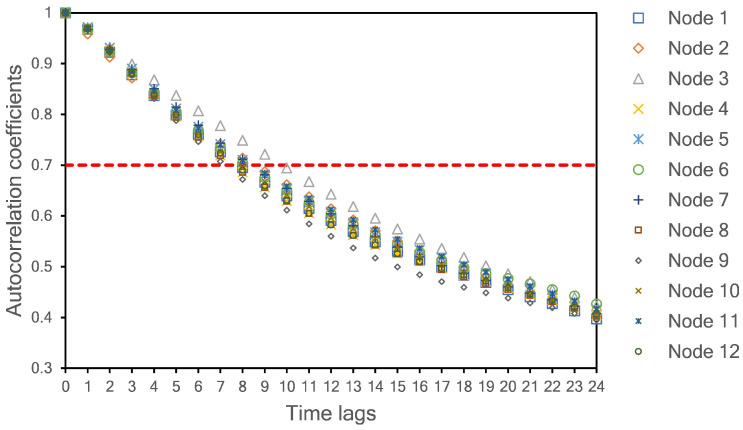
Autocorrelation coefficients for PM_2.5_ concentration with different time lags.

To obtain the RMSE and MAE values shown in Table 3, we used a simple LSTM network as a baseline model before implementing our proposed hybrid model (see Section 4.3). A one-layer LSTM with 15 neurons was selected as a model predictor. The lookback length of the input is determined by calculating the autocorrelation coefficient among the lagged time series of PM_2.5_ data. We set 0.7 as a minimum requirement for high temporal correlation among the lagged data. As shown in Figure 5, eight samples (including time lag = 0) are selected as the length of the input model. At this time lag, all autocorrelation coefficients have values higher than 0.7 for all monitoring sites. Thus, we used the current sample (time lag = 0) and the previous seven samples to predict one sample in the future.

### 4.3. Proposed Model

**Figure 6:**
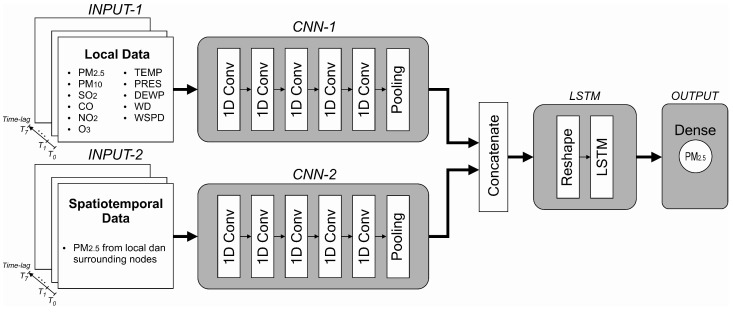
The proposed hybrid CNN-LSTM model. In this model, there are two parallel inputs (INPUT-1 and INPUT-2), one coverts the local node data (processed by CNN-1), and the other contains spatiotemporal dependency of PM_2.5_ data (processed by CNN-2).

In Section 4.2, we implemented a simple, single-layer LSTM model composed of 15 neurons to evaluate model performance based on different input attributes. From this experiment, we can determine which attributes should be fed to the model. In this section, we propose a hybrid model by combining one-dimensional convolutional neural networks (1D CNN) as feature extractors and feeding the output of these CNNs to an LSTM network, as shown in Figure 6.

The proposed model is composed of two inputs, both are formed in a parallel structure. In the first input (INPUT-1), only local (present) node data are collected, whereas, in the second input (INPUT-2), all PM_2.5_ data obtained from local and surrounding nodes are fed. Local node refers to the node where PM_2.5_ is being predicted. Data for INPUT-1 are PM_2.5_, PM_10_, SO_2_, CO, NO_2_, O_3_, temperature, air pressure, dew point, wind direction and wind speed (11 features in total). Eight timesteps (lookback) of these inputs are used to predict one hour of PM_2.5_ in the future. Each batch of inputs is fed to the CNN network, which acts as a feature extractor before entering the LSTM network. After various experiments, we determined the properties of the CNN networks. Both CNN networks (block CNN-1 and CNN-2 in Figure 6) are composed of five convolutional layers and a single average pooling layer. The reshape layer configures the outputs produced by the CNN layers before entering the LSTM network. The same number of neurons are maintained from the previous experiment (15 neurons) with the rectified linear unit (ReLU) activation function. A dense layer with one neuron yields the final prediction. During the training process, the Adam optimiser was used. The properties of each layer are summarised in Table 4.

As explained in Section 4.2, we use eight samples to predict one future sample. To implement deeper convolutional layers as feature extractors to a relatively short data length (in our case, eight samples), we should set small kernel sizes. The length of the next convolutional layer can be calculated using Equation (2). By setting a small value of kernel size *k*, we can get higher output size *o*. Thus, a small kernel size will give more possibilities to operate another convolutional layer in the next step. In our work, we use kernel size equal to 3 for the first and second convolutional layers and kernel size equal to 2 for remaining three convolutional layers. The selected kernel sizes and filters shown in Table 4 are obtained based on our various experiments. Choosing a smaller filter size for each layer will produce a smaller final model size. Thus, it will benefit to our edge device. We found that the filter sizes of 50, 30, 15, 10 and 5 for each convolutional layer in our model produce the best result. We also discovered that the same properties of CNN-1 and CNN-2 yield optimum solutions as feature extractors while maintaining work balance for each input during training and inferencing stages.

### 4.4. Spatiotemporal Dependencies

In this study, both spatial and temporal qualities are studied. The temporal factor is taken into account by selecting time-lag data (lookbacks) as a model input, as discussed in Section 4.2. A time lag equal to zero indicates the current sample. When the time-lag is less than 8, the autocorrelation coefficient is higher than 0.7 for all nodes. This autocorrelation value indicates a high temporal correlation. Therefore, we use eight values for the input length (current measured value plus seven past values).

As mentioned in Section 4.3, in the first input of the model (INPUT-1), temporal dependency of the local node data is covered. The attributes involved for the first input are eight timesteps of PM_2.5_, PM_10_, SO_2_, CO, NO_2_, O_3_, temperature, air pressure, dew point, wind direction and wind speed. We can consider that in INPUT-1, only temporal data are covered. However, in the second input of the model (INPUT-2), both temporal and spatial data of the local and pairing nodes are included. In the second input, we collect only eight timesteps of all PM_2.5_ data (from local and surrounding nodes) and neglect all other environmental and meteorological data. All PM_2.5_ samples from 12 nodes are analysed and the PM_2.5_ correlation coefficients between nodes are calculated. Evaluating the correlation coefficient can indicate the effect of spatial dependency. As shown in Table 5, PM_2.5_ concentrations have a strong correlation (r>0.7) among nodes. A strong correlation implies that there is a high spatial dependency for PM_2.5_ among nodes. Therefore, in this experiment, we include a feature extraction process for the PM_2.5_ concentrations at all neighbouring nodes (data INPUT-2).

**Figure 7:**
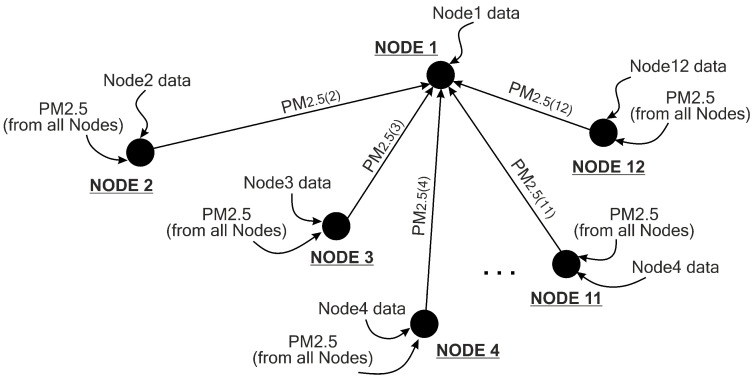
Illustration of spatiotemporal consideration for predicting the value of PM2.5 concentration at Node 1. Nodes 2–12 send their PM_2.5_ data to Node 1. Node 1 uses these PM_2.5_ data as the second input of the proposed model (INPUT-2 in Figure 6), whereas the local data at Node 1 are used as the first input (INPUT-1 in Figure 6). This technique also applies to all other nodes.

Figure 7 depicts the kinds of input data required to forecast the value of PM_2.5_ at a certain node. If we want to forecast the next 1-h value of PM_2.5_ concentration at Node 1, we need to use current pollutant and meteorological samples plus seven previous samples collected by that node (the first input of the proposed model) and collect all PM_2.5_ values from all other nodes (the second input of the proposed model). This scenario also applies to all other nodes.

### 4.5. Deep Learning Data Processing

**Figure 8:**
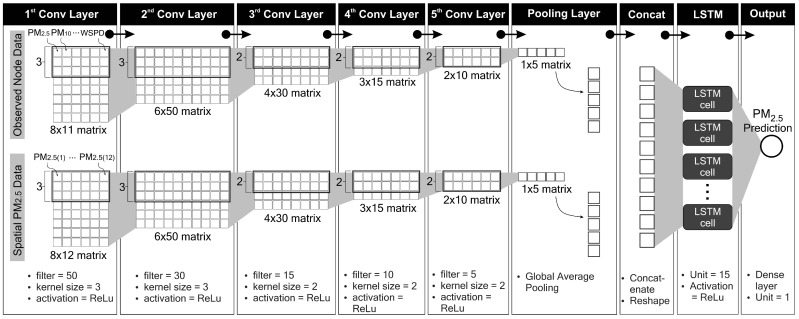
Details of data processing in the proposed deep learning model.

The properties of our proposed deep learning model are summarised in Table 4. In this section, we discuss the internal inference process in our deep learning model. In CNN-1, the eight timesteps of 11 input features form an 8×11 matrix. These 11 features are composed of pollutant and meteorological data (PM_2.5_, PM_10_, SO_2_, CO, NO_2_, O_3_, temperature, air pressure, dew point, wind direction and wind speed). In CNN-2, the eight timesteps of 12 input features form an 8×12 matrix. These 12 features consist of PM_2.5_ concentrations at 12 nodes. According to Equation (2), with a kernel (or feature detector) size of 3 and a stride step of 1, the kernel slides through the input matrix for six steps ((8−3)/1+1=6). With a filter size of 50, the first convolutional layer yields a 6×50 matrix. In the second convolutional layer, the input is now a 6×50 matrix. With a size of 3, the kernel slides along the window for four steps ((6−3)/1+1=4) and produces a 4×30 matrix (since the filter size is 30). The same process applies to all convolutional layers. Thus, the fifth convolutional layer yields a 1×5 matrix. A global average pooling layer behaves as a flattening process. By concatenating both CNN layer outputs, the tensor is ready to enter the LSTM network. The LSTM network consists of 15 cells (or units). Details of the data processing inside an LSTM cell are discussed in Section 3.2. Finally, a single dense layer produces the final result, i.e. our PM_2.5_ prediction. Figure 8 summarises this process.

### 4.6. The Selected Edge Devices

Having evaluated the proposed deep learning model, we now optimise and deploy that model to edge devices. In this work, we utilised the Raspberry Pi, a popular, credit card-sized yet powerful single-board computer developed by the Raspberry Pi Foundation. In recent years, there have been considerable varieties of applications developed using Raspberry Pi boards [48]. We chose two different Raspberry Pi boards: Raspberry Pi 3 Model B+ (RPi3B+) and Raspberry Pi 4 Model B (RPi4B) to show the variation in model performance. The RPi4B is more computationally capable than the RPi3B+. Table 6 shows a feature comparisons between the two boards.

We selected Raspberry Pis since these boards support both TensorFlow and TensorFlow Lite frameworks. Therefore, we can explore wide-range functionalities related to post-training quantisation provided by TensorFlow and demonstrate the performance of both original and quantised models by calculating the model accuracy, the obtained model file sizes and the execution time directly at the edge. Moreover, the Raspberry Pi’s rapid use for research and hobbyist purposes gave rise to many online forums and communities.

## 5. Results and Discussion

### 5.1. Evaluation Scenario

**Figure 9:**
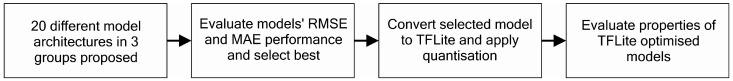
Evaluation scenario used in this work.

The evaluation process in this section can be generally described as follows. We provide 20 different deep learning models and divide these models into three groups. The performance of all models is evaluated based on the attained RMSE and MAE values at all nodes. The best model becomes our proposed model. The TensorFlow file of the proposed model is then converted into a TensorFlow Lite model. In this work, we used TensorFlow version 2.2. Further optimisation is conducted by implementing post-training quantisation of the original TensorFlow model. Finally, the performance of each TensorFlow Lite model is evaluated. The resulted model file size, the execution time and the prediction performance of each TensorFlow Lite model are reported. Figure 9 illustrates this process.

### 5.2. Model Performance

Based on pollutant and meteorological data from the current and the previous 7 h, we predict the short-term PM_2.5_ concentration for 1 h in the future. Model performance was measured based on the obtained RMSE and MAE values evaluated at all nodes. The values of RMSE and MAE were calculated using Equations (9) and (10), respectively. Table 7 summarises the obtained RMSE and MAE of all models, with Node 1 as a representative. The complete result of all nodes is presented in Table A1 and Table A2. We compared our proposed model against several deep learning architectures and proved that our proposed model outperforms other models. Model comparison in Table 7 can be explained as follows:

- *Simple models with local data only* (Group I) take input samples directly without passing them through CNN layers. In these models, the convolutional, pooling, concatenation and reshaping layers are omitted. Thus, the inputs are directly supplied to the RNN, LSTM, GRU or Bidirectional layers. The kinds of inputs used in this architecture are PM_2.5_, PM_10_, SO_2_, CO, NO_2_, O_3_, and meteorological data such as temperature, air pressure, dew point, wind direction and wind speed.
- *Hybrid models with local data only* (Group II) filter input samples through the CNN layers before entering the ANN, RNN, LSTM, GRU or Bidirectional layers. These architectures are hybrid models. In this group, only INPUT-1 and CNN-1 layers are considered, whereas INPUT-2 and CNN-2 layers are neglected. The properties of the CNN layers are described in Table 4. In this case, only PM_2.5_, PM_10_, SO_2_, CO, NO_2_, O_3_ and meteorological data such as temperature, air pressure, dew point, wind direction and wind speed are used as the model inputs, without considering neighbouring PM_2.5_ samples.
- *Hybrid models with spatiotemporal dependency* (Group III) use two inputs (INPUT-1 and INPUT-2), and each input is filtered by CNN layers (CNN-1 and CNN-2). The first input covers the pollutant and meteorological data for the node under consideration, while the second input comprises PM_2.5_ samples from neighbouring nodes. Models in Group III comply with Figure 6, but we vary the LSTM layer with ANN, RNN, GRU or Bidirectional layers.
- Artificial neural network (ANN), recurrent neural network (RNN), long short-term memory (LSTM) and gated recurrent unit (GRU) models in all groups were evaluated and their performances were compared. For fairness, all models in all groups are composed of one hidden layer with 15 neurons (units) inside the layer. At the output layer, there is a dense layer with one neuron to produce the final prediction.
- *Bidirectional layers* are an extension of conventional RNN, LSTM and GRU with two different input directions. First, the input sequence is treated in the usual direction. Second, the input sequence is handled in reverse direction. This scenario can offer additional context to the model and may result in faster and even deeper learning on the input sequence.

As shown in Table 7, we compared 20 different models. We can see that by adding a deeper model (CNN layers) as feature extractor before the predictor (ANN, RNN, LSTM or GRU) will slightly improve models performances. Generally, Group II has better performance than Group I. Adding spatiotemporal considerations along with pollutant and meteorological data as inputs of the model can increase the accuracy. At some nodes, the results can be improved significantly. For example, at Node 1, Groups I and II produce RMSE values between 17 and 19, whereas Group III produces RMSE values between 15 and 17. The best RMSE value was obtained by our proposed model (model no 16 in Table 7), which is 15.322. This RMSE value is better than all other investigated models. For instance, the Bidirectional RNN model in Group I yielded an RMSE value of 19.377, the CNN-LSTM model in Group II produced 17.652, and the CNN-ANN model in Group III returned the RMSE value of 17.160. If we continue to look in more detail to other nodes in Table A1 and Table A2, the PM_2.5_ concentration at Node 11 can be better forecast, not only by our proposed model but also by other investigated models. In contrast, the PM_2.5_ concentration at Node 12 was the hardest to predict as indicated by the higher RMSE and MAE values. For all nodes, our proposed model produced the best performance with error values between 14 and 18 for RMSE, and between 7 and 9 for MAE. The obtained RMSE and MAE values are linearly related. Therefore, by evaluating the RMSE values, we can get an overview of the MAE values.

**Figure 10:**
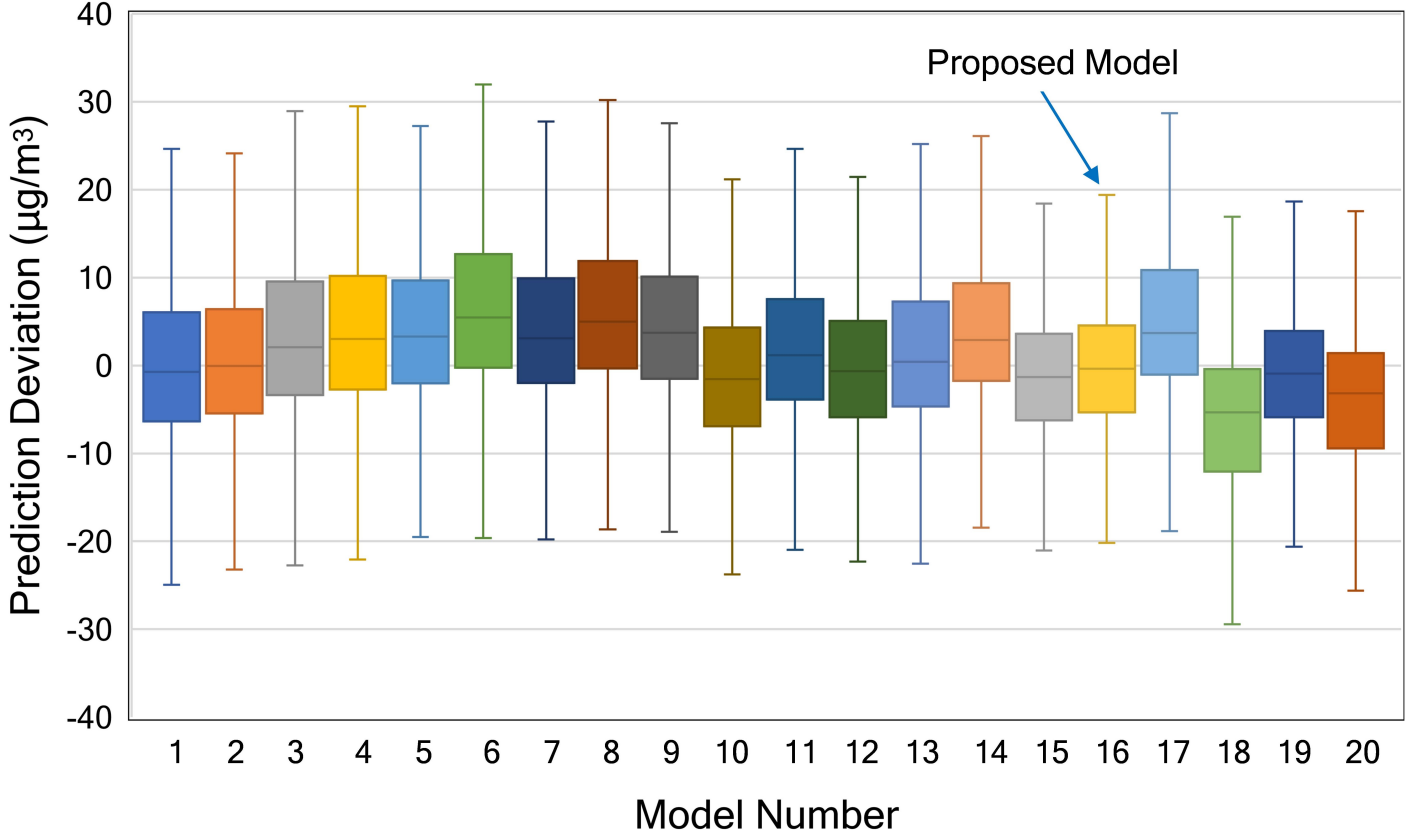
Boxplot of the prediction deviation of all investigated models at Node 1.

Figure 10 shows the boxplot of prediction deviation of all model. The prediction deviation is obtained by subtracting the real value of data test from the predicted values of the model. From the boxplot, we can find information about the variability of the data. The box plot is also useful when we want to compare the distribution between many models. In Figure 10, the solid line in the middle of each box represents the median value. Since the graph represents the prediction deviation between predicted and real data, we prefer this line close to zero. The shorter are the box and whisker, the more centralised are the data. More centralised data indicate that our model is more accurate in predicting the PM_2.5_ data. We also removed the outliers values in order to make the graph more readable. As shown in Figure 10, our proposed model gives the best result as it produces more centralised data and the median value closest to zero.

**Figure 11:**
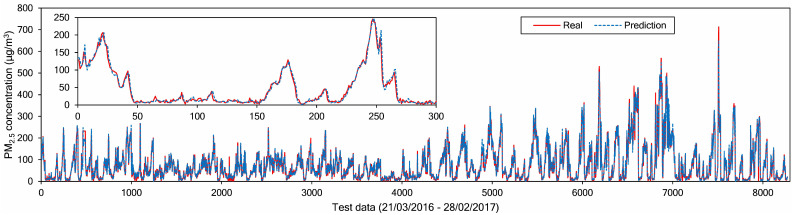
Line plot of real and predicted PM_2.5_ data at Node 1.

To describe model performance more intuitively, Figure 11 shows a line plot between the real and predicted values on the test data at Node 1. The solid line and dashed line indicate the real and predicted values, respectively. There are 8280 samples, collected from 21 March 2016 to 28 February 2017. Overall, the model can capture the fluctuations of future PM_2.5_ values effectively, as shown in Figure 11. The larger errors usually happen when there are spikes in the actual data, whereas for smoother PM_2.5_ data variations, our model forecasts successfully.

**Figure 12:**
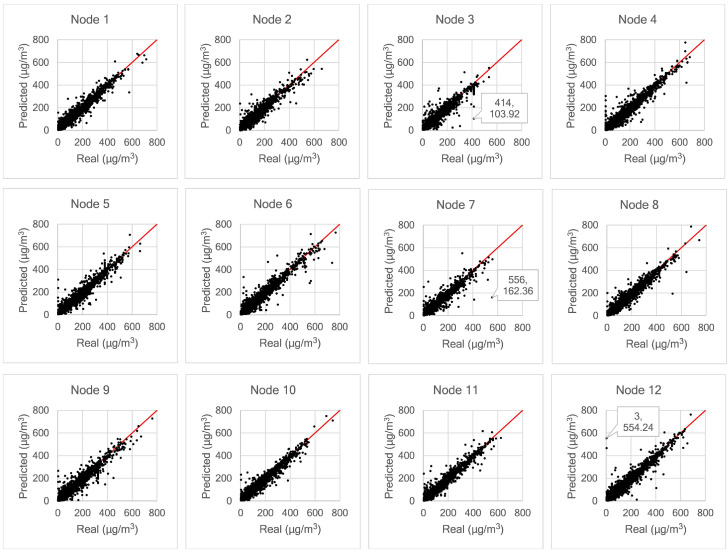
Scatter plots of real and model predicted values of PM_2.5_ at all nodes.

Figure 12 shows scatter plots obtained from all nodes, showing the relationship between real and predicted values; the perfect predictions coincide with the diagonal solid line. Due to the presence of prediction errors the points diverge from this diagonal. All dots mapped below the diagonal solid (ideal) line indicate predictions that are lower than the correct values, while the opposite occurs for points above the ideal line. For example, in the case of Node 3, we observe that more deviations occur below the ideal line. The model predicted 103.92 μg/m^3^, whereas the actual value is 414 μg/m^3^. The same case occurs for Node 7. The model predicted 162.36 μg/m^3^, whereas the real sample is 556 μg/m^3^. Some mispredictions may be due to measurement error, which can be recognised from sudden changes in a sequence of measured samples that are not technically feasible. As shown for Node 12 in Figure 12, there is a significant error in predicting PM_2.5_ data. The model predicted 554.24 μg/m^3^ whereas the measured sensor value is only 3 μg/m^3^ for the labelled point. If we check the dataset at Node 12 in more detail, there had been a sharp drop in the measured value from 621 μg/m^3^ to only 3 μg/m^3^. From 3 μg/m^3^, the measured value then jumps sharply to 144 μg/m^3^. The LSTM network could not recognise these changes. Therefore, there is a significant prediction error at this point.

### 5.3. Model Optimisation for the Edge

After the final model has been trained, the next process is to deploy that model to the edge after optimising it. This Optimisation benefits filesize and computation latency. The initially created model is the TensorFlow model (TF model). From the TF model, we convert our model to a TensorFlow Lite (TFLite model), a lightweight model suitable for edge devices. This TF model can be deployed with or without optimisation, as explained in Section 4.6. We evaluated all possibilities, both with and without optimisation applied. Table 8 summarises the file size comparison between the TF Model and TFLite model. In this case, TFLite has not yet been optimised. The original file size is 318 kilobytes whereas the lite version is 77 kilobytes or four times smaller. File size reduction is an essential step for resource-constrained edges, especially devices with minimal storage available.

**Figure 13:**
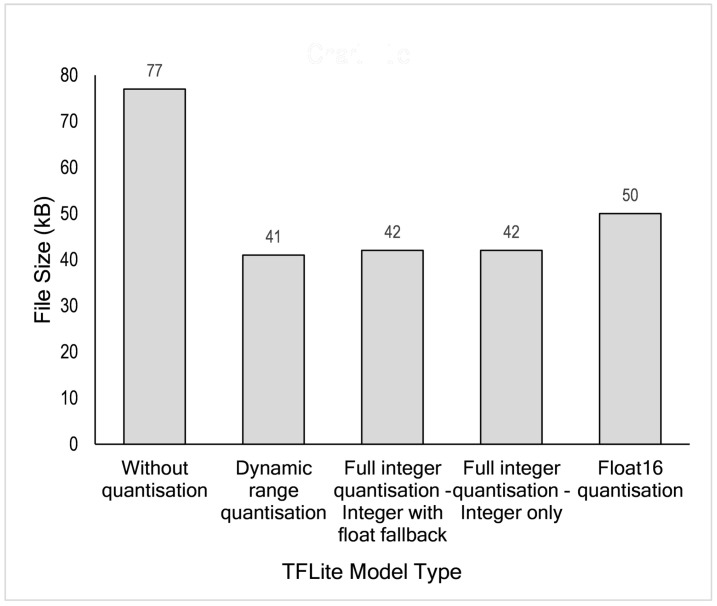
TensorFlow Lite model size comparison.

Further size reduction can be achieved by implementing post-training quantisation. As shown in Figure 13, four different optimisation techniques available in the TensorFlow framework were evaluated for our proposed deep learning model. These techniques are dynamic range quantisation, full integer quantisation with float fallback, integer only quantisation and float16 quantisation. As shown in Table 8 and Figure 13, TFLite model without optimisation/quantisation has a size of 77 kilobytes. Using this model as a reference, an about 47% reduction can be achieved by dynamic range quantisation, about 45% by full integer quantisation and about 35% by float16 quantisation. Based on these results, dynamic range quantisation outperforms other techniques, even though it is just slightly better than full-integer quantisation.

**Figure 14:**
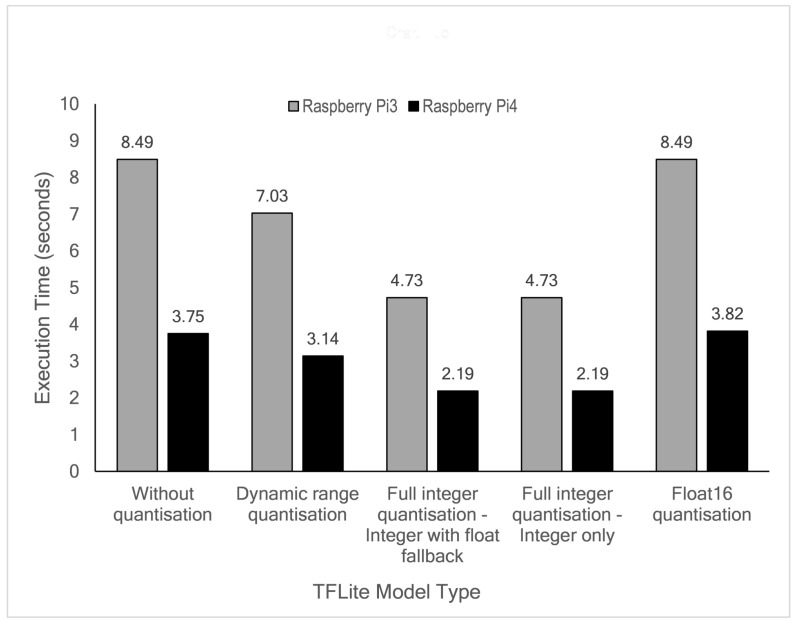
Comparison of TensorFlow Lite execution time for test data.

The time needed for edge devices to predict the available test data was measured. In this study, a total of 8272 hourly samples (data from 21 March 2016 to 28 February 2017) were continuously executed directly at the edge. The experiment results are summarised in Figure 14. As depicted in the figure, the RPi4B board is two times faster than the RPi3B+ board in all quantisation modes. The model with Float16 quantisation does not improve execution time as the latency remains the same with or without quantisation, likely due to the fixed 32-bit floating point datapath on these devices. In this case, the RPi3B+ board needs 8.49 s to execute the complete test, whereas the RPi4B board produces a 0.07 s difference (3.75 and 3.82 s). Even though about 47% size reduction can be achieved by dynamic range quantisation, this mode has minimal execution time improvement. The execution time for this mode is 7.03 and 3.14 s for RPi3B+ and RPi4B, respectively. Full integer quantisation produces the most effective execution time improvement, with latencies of 4.73 and 2.19 s for RPi3B+ and RPi4B, respectively.

**Figure 15:**
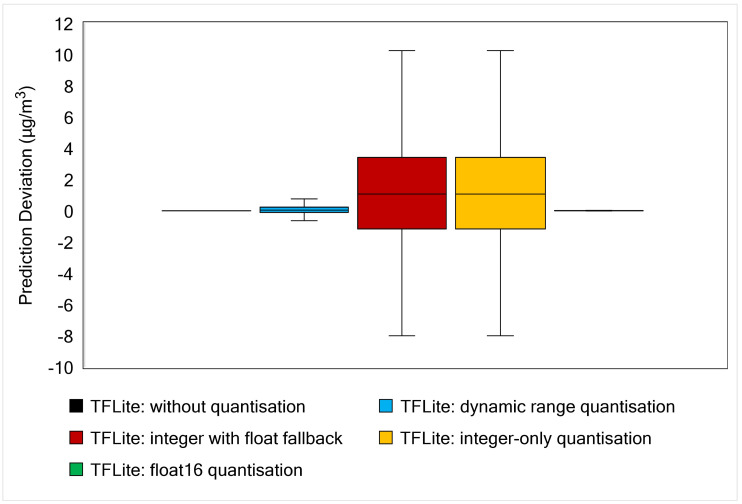
Boxplot of prediction deviation resulted from each TFLite model. The deviation is calculated by subtracting the real values from the predicted values of each TFLite model.

Besides model size and execution time, we must also evaluate model accuracy after applying quantisation. Table A3 in Appendix A shows the details of the RMSE and MAE values for the initial TensorFlow and TensorFlow Lite models. Since the result deviation between the optimised models is very small, we provide a boxplot to present the model performance more intuitively, as shown in Figure 15. This figure provides information about prediction deviation between the result obtained by TFModel and TFLite Model. It is clearly observed that TFLite without quantisation and TFLite with float16 quantisation accuracies are very similar (or produced a very small deviation) to the original TFModel. A slightly longer deviation range is given by TFLite with dynamic range quantisation. Both TFLite integer quantisations give the longest box and whisker range, indicating that these quantisations inferior to other post-quantisation methods in terms of prediction accuracy.

If we are primarily concerned with model accuracy, TFLite without quantisation is a suitable technique. However, it is not the best choice for size reduction and execution time improvement. Dynamic range and float16 quantisations also maintain model accuracy. Dynamic range quantisation produces better model size reduction and execution time than float16 quantisation. Full integer quantisations outperform other TFLite models in terms of model size and latency but slightly reduce model accuracy.

**Figure 16:**
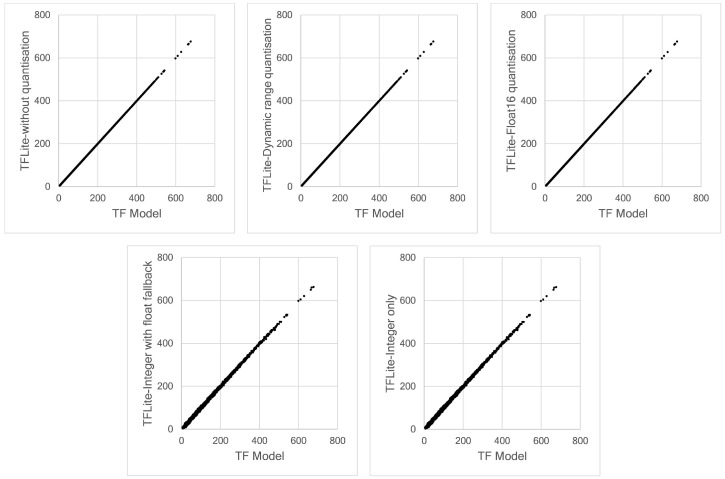
Scatter plot of the prediction data obtained by TensorFlow and TensorFlow Lite models.

To examine the correspondence between our TensorFlow Lite models and the initial TensorFlow model intuitively, we can compare these models using scatter plots, as shown in Figure 16. This figure presents the result at only Node 1. However, the same behaviour occurred for all nodes. The results obtained by the TFLite without quantisation, dynamic range quantisation and float16 quantisation match the results predicted by the initial TensorFlow model, as demonstrated by the a smooth straight-line pattern. We can also observe the same effect in Figure 15. A larger deviation is produced by full integer quantisation models, both integer with fallback and full integer quantisations. The straight-line pattern is more scattered and lead to a conclusion that that full integer quantisation impacts model accuracy, even with a very small deviation.

## 6. Conclusions

Edge computing brings computation closer to data sources (edge) and can be a solution for latency, privacy and scalability issues faced by a cloud-based system. It is also possible to embed intelligence at the edge, which can be enabled by Machine Learning algorithms. Deep Learning, a subset of ML, can be implemented at the edge. In this work, we propose a hybrid deep learning model composed of 1D Convolutional Neural Network and Long-Short Term Memory (CNN-LSTM) networks to predict a short-term hourly PM_2.5_ concentration at 12 different nodes. The results showed that our proposed model outperformed other possible deep learning models, evaluated by calculating RMSE and MAE errors at each node. To implement an efficient model for edge devices, we applied four different post-quantisation techniques provided by TensorFlow Lite framework: dynamic range quantisation, float16 quantisation, integer with float fallback quantisation and full integer-only quantisation. Dynamic range and float16 quantisations maintain model accuracy but did not improve latency significantly. Meanwhile, full integer quantisation outperformed other TFLite models in terms of model size and latency but slightly reduced model accuracy. The targeted edge devices in our work are the Raspberry Pi 3 Model B+ and Raspberry Pi 4 Model B boards. Technically, the Raspberry Pi 4 demonstrated lower latency due to the more capable processor.

In the future, we plan to develop this work further by offloading model computation for multiple nodes to a gateway device, thereby allowing the sensor nodes to be extremely lightweight. We would also like to explore methods for efficient sharing of a gateway deep learning model by multiple nodes. Finally, we would like to explore how models can be evolved on these edge devices. 

## Figures and Tables

**Table 1:** Summary of the works related to air quality prediction. We studied current research trend on deep learning application and extend this body of work around deep learning models by analysing the deployment of these models to edge device. In the last row of the table, we stated our work contribution.

Reference	Proposed Model	Target Prediction	Model Optimisation	Note
[18]	EWT-MAEGA-NARX	PM_2.5_, SO_2_, NO_2_, and CO	No	Combining the EWT, MAEGA and NARX neural networks for multi-step air pollutant predictions
[26]	LSTM	CO, Average NO_2_, Average O_3_, Average PM_10_, Average SO_2_, Average Plantago, Average Poaceae	No	Combining different groupings of inputs for the LSTM network. Choosing a single but comprehensive model rather than multiple individual models.
[27]	LSTM Extended	PM_2.5_	No	Adding auxiliary inputs (meteorological data, month of year, and hour of day) for LSTM network
[28]	LSTM and Deep Autoencoder	PM_2.5_, PM_10_	No	Varying the input batch size and recording the total average of the model performances
[29]	LSTM-based MMSL	PM_2.5_, CO, NO_2_, O_3_, SO_2_	No	Considering spatiotemporal factors
[30]	HighAir Framework	Air Quality Index (AQI), Points of Interest (POI), weather data	No	Using graph neural network based on encoder-decoder architecture. Both encoder and decoder consist of LSTM network
[31]	Geo-LSTM	PM_2.5_	No	Implementing geo-layer to integrate the spatial-temporal correlation from other monitoring stations
[32]	An ensemble LSTM	PM_2.5_	No	Performing 3 steps: ensemble empirical mode decomposition (EEMD), LSTM, inverse EEMD
[33]	LSTM-FC	PM_2.5_	No	Using LSTM-based temporal simulator and NN-based spatial combinatory
[34]	Graph CNN-LSTM	PM_2.5_	No	Implementing graph signal for spatial dependency modelling
[35]	Deep-AIR	PM_2.5_, PM_10_, NO_2_, CO, O_3_	No	Combining CNN-LSTM networks, using ResNet to let the model learn large information
[36]	CNN-LSTM	PM_2.5_	No	Using CNN to eliminate redundancy and obtain the features, using LSTM to predict the time-series
[37]	CNN-LSTM	PM_2.5_	No	Using 1D CNN for feature extraction and the LSTM layer for prediction
[38]	CBGRU	PM_2.5_	No	Using 2 layer CNN and Bidirectional GRU
[39]	LSTM	PM_2.5_	No	Using cloud server node with AI technology
Our work	CNN-LSTM	PM_2.5_	Yes	Implementing post-training quantisation for model optimisation. Deploying deep learning model on edge devices

**Table 2:** Correlation coefficients (*r*) among attributes at Node 1. The value of *r* is obtained by applying Equation (13) between corresponding attributes in the dataset.

Attribute	PM_2.5_	PM_10_	SO_2_	NO_2_	CO	O_3_	TEMP	PRES	DEWP	RAIN	WD	WSPD
PM_2.5_	1	0.87	0.49	0.67	0.76	−0.15	−0.09	−0.02	0.15	−0.01	−0.19	−0.27
PM_10_	0.87	1	0.47	0.65	0.65	−0.12	−0.07	−0.05	0.09	−0.02	−0.12	−0.17
SO_2_	0.49	0.47	1	0.44	0.57	−0.22	−0.36	0.23	−0.29	−0.04	−0.12	−0.11
NO_2_	0.67	0.65	0.44	1	0.66	−0.46	−0.17	0.04	0.12	−0.03	−0.24	−0.48
CO	0.76	0.65	0.57	0.66	1	−0.32	−0.37	0.24	−0.12	−0.01	−0.22	−0.25
O_3_	−0.15	−0.12	−0.22	−0.46	−0.32	1	0.58	−0.42	0.30	0.03	0.21	0.33
TEMP	−0.09	−0.07	−0.36	−0.17	−0.37	0.58	1	−0.83	0.83	0.04	0.05	0.01
PRES	−0.02	−0.05	0.23	0.04	0.24	−0.42	−0.83	1	−0.78	−0.06	−0.02	0.09
DEWP	0.15	0.09	−0.29	0.12	−0.12	0.30	0.83	−0.78	1	0.08	−0.13	−0.33
RAIN	−0.01	−0.02	−0.04	−0.03	−0.01	0.03	0.04	−0.06	0.08	1	−0.01	0.00
WD	−0.19	−0.12	−0.12	−0.24	−0.22	0.21	0.05	−0.02	−0.13	−0.01	1	0.31
WSPD	−0.27	−0.17	−0.11	−0.48	−0.25	0.33	0.01	0.09	−0.33	0.00	0.31	1

**Table 3:** Model performance based on different input attributes for Node 1.

Input Features	Number of Inputs	RMSE	MAE
All	12	17.704	10.017
Without rain	11	17.363	9.807
Without rain and pressure	10	18.168	10.268
Without rain, pressure and temperature	9	17.638	9.937

**Table 4:** Hybrid CNN-LSTM network properties of the proposed model. CNN-1 and CNN-2 have the same convolutional layer properties.

Layer	Properties
1^st^ Convolutional	filter = 50, kernel size = 3, activation = ReLU
2^nd^ Convolutional	filter = 30, kernel size = 3, activation = ReLU
3^rd^ Convolutional	filter = 15, kernel size = 2, activation = ReLU
4^th^ Convolutional	filter = 10, kernel size = 2, activation = ReLU
5^th^ Convolutional	filter = 5, kernel size = 2, activation = ReLU
Pooling	global average pooling
Reshape	reshape ((1,15))
LSTM	units = 15, activation = ReLU
Dense	units = 1

**Table 5:** PM_2.5_ coefficient correlation (*r*) for all nodes. The value of *r* is obtained by using Equation (13) to the PM_2.5_ between corresponding two nodes. Data from 01 March 2013 to 28 February 2017. A strong correlation implies that there is a spatial dependency for particulate matter data. Therefore, when predicting PM_2.5_ data at a certain node, we should consider PM_2.5_ values at other nodes.

	Node1	Node2	Node3	Node4	Node5	Node6	Node7	Node8	Node9	Node10	Node11	Node12
Node1	1	0.84	0.83	0.95	0.96	0.89	0.83	0.94	0.88	0.93	0.93	0.91
Node2	0.84	1	0.90	0.81	0.83	0.84	0.84	0.80	0.80	0.80	0.86	0.78
Node3	0.83	0.90	1	0.80	0.83	0.84	0.85	0.79	0.81	0.79	0.85	0.77
Node4	0.95	0.81	0.80	1	0.97	0.89	0.82	0.95	0.88	0.96	0.93	0.93
Node5	0.96	0.83	0.83	0.97	1	0.92	0.84	0.94	0.88	0.95	0.95	0.94
Node6	0.89	0.84	0.84	0.89	0.92	1	0.85	0.87	0.85	0.89	0.93	0.88
Node7	0.83	0.84	0.85	0.82	0.84	0.85	1	0.80	0.89	0.81	0.84	0.79
Node8	0.94	0.80	0.79	0.95	0.94	0.87	0.80	1	0.87	0.94	0.91	0.92
Node9	0.88	0.80	0.81	0.88	0.88	0.85	0.89	0.87	1	0.87	0.87	0.86
Node10	0.93	0.80	0.79	0.96	0.95	0.89	0.81	0.94	0.87	1	0.92	0.95
Node11	0.93	0.86	0.85	0.93	0.95	0.93	0.84	0.91	0.87	0.92	1	0.90
Node12	0.91	0.78	0.77	0.93	0.94	0.88	0.79	0.92	0.86	0.95	0.90	1

**Table 6:** Raspberry Pi 3 Model B+ and Raspberry Pi 4 Model B feature comparisons.

	RPi3B+	RPi4B
SoC	Broadcom BCM2837B0	Broadcom BCM2711
CPU	Quad-core Cortex-A53 (ARMv8) 64-bit @1.4 GHz	Quad-core Cortex-A72 (ARMv8) 64-bit @1.5 GHz
GPU	Broadcom VideoCore IV @250 MHz	Broadcom VideoCore VI @500 MHz
FPU	VFPv4 + NEON	VFPv4 + NEON
RAM	1 GB LPDD*R*^2^ SDRAM	2 GB LPDDR4 SDRAM (used in this experiment)
Storage	microSD card	microSD card
Power ratings	Idle: 459 mA (2.295 Watt), Maximum: 1.13 A (5.661 Watt)	Idle: 600 mA (3 Watt), Maximum: 1.25 A (6.25 Watt)

**Table 7:** Comparison of RMSE and MAE values for PM_2.5_ prediction using different model architectures calculated for Node 1. Twelve different model architectures are proposed and categorised into three groups. The best model for each group is indicated in bold. The proposed model in this work belongs to Group III (Model 16). In Group III, both spatial and temporal dependencies are considered. Our proposed model yields the lowest RMSE and MAE values. Detailed results for all nodes are shown in Table A1 and Table A2.

No.	Model Type	RMSE	MAE
	*Simple models with local data only (Group I)*	
1	RNN	18.485	10.636
2	**LSTM**	**17.786**	**10.230**
3	GRU	18.367	10.664
4	Bidirectional RNN	19.377	12.257
5	Bidirectional LSTM	18.016	10.427
6	Bidirectional GRU	18.603	10.944
	*Hybrid models with local data only (Group II)*	
7	CNN-ANN	17.757	10.321
8	CNN-RNN	18.227	10.906
9	CNN-LSTM	17.652	10.203
10	**CNN-GRU**	**17.244**	**9.552**
11	CNN-Bidirectional RNN	17.334	10.001
12	CNN-Bidirectional LSTM	17.344	10.054
13	CNN-Bidirectional GRU	17.462	10.486
	*Hybrid models with spatiotemporal dependency (Group III)*	
14	CNN-ANN	17.160	10.307
15	CNN-RNN	15.672	9.162
16	**CNN-LSTM (Proposed Model)**	**15.268**	**8.778**
17	CNN-GRU	17.169	9.665
18	CNN-Bidirectional RNN	17.365	10.443
19	CNN-Bidirectional LSTM	15.643	8.853
20	CNN-Bidirectional GRU	16.089	9.512

**Table 8:** TensorFlow and TensorFlow Lite file size comparison.

Properties	TF Model	TFLite Model
File size (kB)	318	77

## Data Availability

Not applicable.

## Abbreviations

The following abbreviations are used in this manuscript:

1D CNN	One-Dimensional Convolutional Neural Network
ANN	Artificial Neural Network
DL	Deep Learning
GRU	Gated Recurrent Units
IoT	Internet of Things
LSTM	Long Short-Term Memory
MAE	Mean Absolute Error
ML	Machine Learning
PM	Particulate Matter
RMSE	Root Mean Square Error
RNN	Recurrent Neural Network
RPi3B+	Raspberry Pi Model 3B+
RPi4B	Raspberry Pi Model 4B

## Appendix A

In this section, we report the RMSE and MAE of the predicted values of all nodes. Table A1 and Table A2 show the RMSE and MAE values, respectively. Table A3 shows the effect of quantisation techniques on the RMSE and MAE values.

**Table A1:** Comparison of RMSE values for PM_2.5_ prediction using different model architectures for all nodes.

No.	Architectures	Node1	Node2	Node3	Node4	Node5	Node6	Node7	Node8	Node9	Node10	Node11	Node12
	*Simple Models Group I*												
1	RNN	18.485	17.961	19.356	20.655	19.332	21.088	17.186	19.560	21.046	17.892	20.920	23.149
2	LSTM	17.786	18.203	19.802	19.775	19.186	19.434	17.620	18.208	21.143	17.586	17.946	21.180
3	GRU	18.367	18.353	18.475	22.143	20.578	20.459	17.449	19.471	20.914	18.435	19.267	22.257
4	Bidirectional RNN	19.377	17.383	17.799	20.703	18.864	20.737	17.522	18.740	20.125	17.450	18.442	21.996
5	Bidirectional LSTM	18.016	17.084	18.967	20.806	18.829	19.563	17.547	19.041	19.299	17.144	17.335	22.520
6	Bidirectional GRU	18.603	17.606	17.650	21.290	19.275	19.339	16.899	18.443	18.764	17.052	17.138	21.489
	*Hybrid Models Group II*												
7	CNN-ANN	17.757	16.838	17.841	19.752	19.207	19.793	17.174	18.777	20.364	17.635	17.041	21.537
8	CNN-RNN	18.227	16.813	17.445	20.021	18.420	19.303	16.952	18.418	18.686	16.713	17.018	21.492
9	CNN-LSTM	17.652	16.801	18.387	19.743	19.184	19.228	17.261	18.242	18.663	17.040	17.209	21.160
10	CNN-GRU	17.244	16.742	17.733	19.667	19.759	20.897	17.001	18.391	19.652	16.611	17.081	21.818
11	CNN-Bidirectional RNN	17.334	16.804	17.571	19.514	18.520	19.083	18.062	18.109	20.697	16.908	16.962	21.421
12	CNN-Bidirectional LSTM	17.344	17.981	20.118	20.267	19.640	19.071	16.862	18.058	18.427	16.676	17.071	21.667
13	CNN-Bidirectional GRU	17.462	17.518	20.038	21.214	18.642	19.098	16.800	19.131	18.497	17.230	16.944	21.268
	*Hybrid Models Group III*												
14	CNN-ANN	17.160	17.661	18.493	18.074	17.282	18.598	19.235	17.969	18.196	17.018	17.705	19.134
15	CNN-RNN	15.672	17.159	18.377	18.135	16.933	18.262	16.135	18.596	20.215	16.053	15.981	19.494
16	CNN-LSTM (proposed model)	15.268	15.710	17.082	17.706	16.557	17.743	15.493	16.172	17.920	14.894	14.951	18.962
17	CNN-GRU	17.169	16.136	20.252	18.900	17.034	20.692	18.166	18.327	18.897	16.250	17.031	19.098
18	CNN-Bidirectional RNN	17.365	18.182	18.229	19.658	17.591	18.301	16.045	16.818	18.068	15.513	15.154	19.426
19	CNN-Bidirectional LSTM	15.643	16.647	18.085	17.941	17.574	18.544	15.845	16.584	19.187	15.530	16.423	19.038
20	CNN-Bidirectional GRU	16.089	16.855	19.121	18.193	17.269	19.350	15.789	16.545	18.134	15.874	15.785	19.267

**Table A2:** Comparison of MAE values for PM_2.5_ prediction using different model architectures for all nodes.

No.	Architectures	Node1	Node2	Node3	Node4	Node5	Node6	Node7	Node8	Node9	Node10	Node11	Node12
	*Simple Models Group I*												
1	RNN	10.636	10.123	10.206	11.412	10.904	12.043	8.636	11.080	11.571	10.686	12.403	12.139
2	LSTM	10.230	10.710	10.376	10.670	10.906	11.174	8.786	10.499	11.829	10.880	10.523	11.078
3	GRU	10.664	10.769	9.881	12.505	11.635	11.57	8.880	11.548	11.977	11.053	11.183	11.947
4	Bidirectional RNN	12.257	9.898	9.349	11.871	10.743	11.887	9.040	10.571	11.154	10.587	11.144	11.560
5	Bidirectional LSTM	10.427	9.462	10.335	11.710	10.584	11.098	8.764	10.826	10.461	10.357	9.765	11.710
6	Bidirectional GRU	10.944	10.345	9.429	13.051	10.924	10.665	8.338	10.171	10.124	10.404	9.658	11.348
	*Hybrid Models Group II*												
7	CNN-ANN	10.321	9.387	9.671	10.679	11.351	11.333	8.714	11.267	11.466	10.757	9.385	11.558
8	CNN-RNN	10.906	9.959	8.888	11.311	10.303	10.746	8.306	10.419	9.961	10.043	9.615	11.123
9	CNN-LSTM	10.203	9.365	9.727	10.582	11.176	10.571	8.914	10.027	10.407	10.084	9.612	11.216
10	CNN-GRU	9.552	9.423	9.213	10.622	11.771	12.006	8.420	10.410	11.313	10.077	9.492	11.52
11	CNN-Bidirectional RNN	10.001	9.443	9.383	10.274	10.562	10.312	9.750	10.179	12.417	10.072	9.683	10.833
12	CNN-Bidirectional LSTM	10.054	10.439	11.576	11.778	12.542	10.670	8.313	10.245	9.858	9.895	9.700	11.282
13	CNN-Bidirectional GRU	10.486	9.923	11.638	12.638	10.590	10.440	8.273	11.383	10.290	10.684	9.466	11.210
	*Hybrid Models Group III*												
14	CNN-ANN	10.307	10.351	9.221	9.872	10.168	10.446	12.906	10.637	10.245	10.564	10.191	9.985
15	CNN-RNN	9.162	9.602	8.998	10.106	9.510	10.069	8.326	11.068	12.817	10.055	9.543	10.295
16	CNN-LSTM (proposed model)	8.778	8.873	8.803	9.653	9.228	9.590	7.607	9.116	9.588	8.993	8.486	9.641
17	CNN-GRU	9.665	8.967	11.431	11.118	9.981	11.852	11.023	10.252	10.838	10.068	10.274	9.878
18	CNN-Bidirectional RNN	10.443	10.265	9.054	11.354	9.978	10.115	8.286	9.676	9.709	9.213	8.527	10.269
19	CNN-Bidirectional LSTM	8.853	9.297	9.657	9.763	10.177	9.746	7.980	10.095	10.167	9.203	10.149	9.815
20	CNN-Bidirectional GRU	9.512	9.644	9.838	10.037	9.331	10.863	7.977	9.665	9.780	9.786	9.143	10.111

**Table A3:** Effect of quantisation techniques on RMSE and MAE values.

	Node1	Node2	Node3	Node4	Node5	Node6	Node7	Node8	Node9	Node10	Node11	Node12
*Initial TensorFLow model*												
RMSE	15.268	15.710	17.082	17.706	16.557	17.743	15.493	16.172	17.920	14.894	14.951	18.962
MAE	8.778	8.873	8.803	9.653	9.228	9.590	7.607	9.116	9.588	8.935	8.486	9.641
*TFLite—without quantisation*												
RMSE	15.268	15.710	17.082	17.706	16.557	17.743	15.493	16.172	17.920	14.894	14.951	18.962
MAE	8.778	8.873	8.803	9.653	9.228	9.590	7.607	9.116	9.588	8.935	8.486	9.641
*TFLite—dynamic range quantisation*												
RMSE	15.270	15.710	17.116	17.679	16.580	17.741	15.490	16.189	17.906	14.905	14.994	18.974
MAE	8.784	8.872	8.844	9.639	9.236	9.592	7.611	9.129	9.604	8.943	8.550	9.678
*TFLite—integer with float fallback*												
RMSE	15.704	16.102	17.506	18.108	17.032	18.167	15.694	16.536	18.758	15.467	15.216	19.690
MAE	9.418	9.420	9.562	10.425	10.004	10.394	8.081	9.674	10.792	9.694	9.048	10.785
*TFLite—integer-only quantisation*												
RMSE	15.704	16.102	17.506	18.108	17.032	18.167	15.694	16.536	18.758	15.467	15.216	19.690
MAE	9.418	9.420	9.562	10.425	10.004	10.394	8.081	9.674	10.792	9.694	9.048	10.785
*TFLite—float16 quantisation*												
RMSE	15.268	15.708	17.082	17.707	16.557	17.742	15.494	16.171	17.920	14.896	14.952	18.962
MAE	8.777	8.871	8.803	9.654	9.228	9.589	7.607	9.115	9.588	8.936	8.486	9.642
